# P-1141. Prevalence of Coagulase-negative Staphylococcus Bloodstream Infections According to Blood Culture Collection

**DOI:** 10.1093/ofid/ofaf695.1335

**Published:** 2026-01-11

**Authors:** Beatriz Arns, Flávia R Brust, Daniel Sganzerla, Mateus Swarovsky Helfer, Vlademir V Cantarelli, Alexandre Zavascki

**Affiliations:** Hospital Moinhos de Vento, Porto Alegre, Rio Grande do Sul, Brazil; Health Security Partners, ´Porto Alegre, Rio Grande do Sul, Brazil; Inova Medical Research, Porto Alegre, Rio Grande do Sul, Brazil; Hospital de Clinicas de Porto Alegre, Porto Alegre, Rio Grande do Sul, Brazil; Federal University of Health Sciences of Porto Alegre, Cachoeirinha, Rio Grande do Sul, Brazil; Hospital de Clínicas de Porto Alegre, Porto Alegre, Rio Grande do Sul, Brazil

## Abstract

**Background:**

Differentiating bloodstream infections (BSI) from commensal contamination remains challenging. For coagulase-negative *Staphylococcus* (CoNS), confirming BSI typically requires multiple blood culture (BC) sets, which is often not feasible in low- and middle-income countries. This study assessed how CoNS BSI prevalence estimates differ when using only one BC set versus two or more BC sets with commonly documented signs/symptoms of infection.

**Methods:**

We conducted a retrospective, cross-sectional study at a tertiary hospital in Brazil, reviewing CoNS-positive BCs collected from Jun 2020 to Dec 2022. Adult patients (≥18 years old) with two or more BC sets collected within 24 hours and at least one positive for CoNS were included. A reference group was defined by two or more CoNS positive BCs plus signs/symptoms (documented fever, chills or hypotension). Four simulated single-BC groups were created: Group A (central line sets), Group B (peripheral sets), and Groups C and D (random samples from the full dataset)(Figure 1). In simulated groups, we applied the Global Action in Healthcare Network (GAIHN) “BSI-B” definition (one CoNS positive BC plus documented signs/symptoms). We calculated relative frequencies and 95% confidence intervals.

**Results:**

Of a total of 986 episodes, 119 (12%) had two or more positive BCs for CoNS. Of these, 102 (10%) met BSI criteria. In simulated single-BC groups, 44-46% had one positive BC for CoNS, and 37-39% met “BSI-B” criteria, yielding prevalence ratios of 3.6-3.8 (Figure 2). These findings were consistent across groups, regardless of BC collection site.

**Conclusion:**

In settings where only one BC can be collected, using definitions that rely on a single positive CoNS BC plus symptoms may overestimate BSI prevalence by more than threefold. Conversely, requiring two positive BC may underestimate the true burden when multiple sets are not feasible. Pragmatic readily available criteria are needed to help distinguish true CoNS BSI from contamination in single-BC scenarios, especially in low-resource settings.

**Disclosures:**

All Authors: No reported disclosures

